# Behring in the Baby Act

**Published:** 1898-11

**Authors:** 


BEHRING IN THE BABY ACT.—Prof. Emil Behring, the alleged discoverer of diphtheria antitoxin,—the patentee of the process of making the serum, has been roundly scored by the medical press all over the world for "selling his birthright for a mess of potage," for sinking the high professional character he has borne as a scientist, in the commercial man "on the make," and no where else more unmercifully than in America. This is rather a rude awakening of the American medical profession, or rather, of the flunky element of them, who have, heretofore, been ever ready to fall down and worship everybody in Germany called a "scientist," and to laud everything made or discovered in Germany. The mask has been thrown off, and this erstwhile big Injun scientist stands revealed in the nakedness of a common mortal—*on the make.* Good! It is to be hoped that these fellows; i. e., these flunkies, will be able now to see some merit in things of American origin, and be no longer held in bondage to the German pretensions.

The press put it to Mr. Behring in so strong a light; laid it on so long and so hard that this imperturbable and thick skinned Herr was driven, it seems, to a sort of a defense—the more the fool,—an alleged justification of his unrighteous act in patenting the outcome of the ideas of others,—jointly, we admit, with his own, and behold—he pleads the baby act; he was poor; needed money. His own government did not "take stock" in his discovery, as he says,—the French government did in Pasteur's investigations. The French, he says, subscribed liberal sums to enable Pasteur to carry on his laboratory work, which has enriched the world in scientific knowledge, and no part of it more than sunny France; while Herr Behring had to borrow money, even to live on, he says. He would probably have said, had it not been so thread bare, that "a prophet is not without honor save in his own country." Rats! He might have said, and perhaps with some truth, that he had found that "honor" in his own country was without "profit;" at any rate—he swapped the honor for the profit He pleads further, that he is released from that indefinable instinct understood to be meant by *noblesse oblige,*—being no longer a "physician!" That having given up the title and pursuit of *physician*—in order to make a living,—he is no longer bound by the code of medical ethics! Poor devil; he deserves rather our pity than our contempt; we should reserve the contempt for our vacillating patent office people who, five times *wouldn't,* and then finally would, issue a patent,—under pressure of—what?

